# Deep metagenomic sequencing unveils novel SAR202 lineages and their vertical adaptation in the ocean

**DOI:** 10.1038/s42003-024-06535-5

**Published:** 2024-07-12

**Authors:** Changfei He, Daniel Fucich, Ana Sosa, Hualong Wang, Jinjun Kan, Jihua Liu, Yongle Xu, Nianzhi Jiao, Michael Gonsior, Feng Chen

**Affiliations:** 1 State Key Laboratory of Marine Environmental Science, College of Ocean and Earth Sciences, Carbon Neutral Innovation Research Center and Fujian Key Laboratory of Marine Carbon Sequestration, Xiamen University, Xiamen, 361102 PR China; 2 Institute of Marine Science and Technology, Shandong University, Qingdao, 266237 China; 3 Institute of Marine and Environmental Technology, University of Maryland Center for Environmental Science, Baltimore, MD 21202 USA; 4 College of Marine Life Sciences, Frontiers Science Center for Deep Ocean Multispheres and Earth System, and Key Lab of Polar Oceanography and Global Ocean Change, Ocean University of China, Qingdao, China; 5 Microbiology Division, Stroud Water Research Center, Avondale, PA 19311 USA; 6 Chesapeake Biological Laboratory, University of Maryland Center for Environmental Science, Solomons, MD 20783 USA

**Keywords:** Water microbiology, Microbial ecology

## Abstract

SAR202 bacteria in the Chloroflexota phylum are abundant and widely distributed in the ocean. Their genome coding capacities indicate their potential roles in degrading complex and recalcitrant organic compounds in the ocean. However, our understanding of their genomic diversity, vertical distribution, and depth-related metabolisms is still limited by the number of assembled SAR202 genomes. In this study, we apply deep metagenomic sequencing (180 Gb per sample) to investigate microbial communities collected from six representative depths at the Bermuda Atlantic Time Series (BATS) station. We obtain 173 SAR202 metagenome-assembled genomes (MAGs). Intriguingly, 154 new species and 104 new genera are found based on these 173 SAR202 genomes. We add 12 new subgroups to the current SAR202 lineages. The vertical distribution of 20 SAR202 subgroups shows their niche partitioning in the euphotic, mesopelagic, and bathypelagic oceans, respectively. Deep-ocean SAR202 bacteria contain more genes and exhibit more metabolic potential for degrading complex organic substrates than those from the euphotic zone. With deep metagenomic sequencing, we uncover many new lineages of SAR202 bacteria and their potential functions which greatly deepen our understanding of their diversity, vertical profile, and contribution to the ocean’s carbon cycling, especially in the deep ocean.

## Abstract (web summary)

The authors apply deep metagenomic sequencing to analyze six depth samples at the BATS station. They identify novel SAR202 groups/subgroups in the ocean to understand their vertical distribution, metabolic diversity, unique ecological niches.

## Introduction

SAR202 bacteria were initially discovered in the Bermuda Atlantic time series (BATS) study site^1^ and subsequently in the deep Atlantic and Pacific oceans^2^ through clone library analysis of the prokaryotic 16 S rRNA gene. Later studies showed that SAR202 could comprise about 10% of all plankton cells in the dark ocean^3^ and contribute up to 30% of the bathypelagic microbial community^4–6^. SAR202 is widely distributed in natural environments such as pelagic seawater, marine sediment, soil, and deep subsurface terrestrial habitats^7–11^.

Although SAR202 bacteria are abundant and ubiquitously distributed in the ocean, they have not been cultivated in the laboratory until recently^12^. In the past, our understanding of their potential biogeochemical role mainly relied on assembled genomes based on metagenomics or single-cell metagenomics^13–16^. SAR202 bacteria are very complex in terms of their taxonomic structure and metabolic properties^13,15^. Paralogous flavin-dependent monooxygenase (FMNO) genes were found to be mainly enriched in the SAR202 group III, in some cases exceeding 100 genes per genome, and were later extended to other groups^13,15^. Many oxidative enzymes appear to play complementary roles in the degradation of complex organic carbon such as aromatic compounds^13^. The genomes of SAR202 bacteria contain genes for C1 oxidation such as formaldehyde and formate dehydrogenase, as well as genes for fatty acid beta-oxidation, suggesting that SAR202 bacteria have the potential to harvest carbon and energy from diverse organic molecules such as from simple C1 molecules to complex cyclic compounds^13,15,17^. SAR202 genomes have shown the potential to metabolize multiple organosulfur compounds, many appear to be sulfite-oxidizers and are predicted to play a major role in sulfur turnover in the ocean^14,18^. SAR202 bacteria encode genes for ammonia assimilation and utilization of other potential nitrogen sources such as hypotaurine and taurine in the deep sea^14,16^.

SAR202 bacteria have been divided into seven groups (I to VII) based on the genomic phylogeny, with six subgroups (Ia-c and IIIa-c) in groups I and III^13,15,18^. These different groups and subgroups occupy different niches of the water column in the ocean^15,16^. Current culturable SAR202 bacterial strains are all from subgroup Ia, only representing a small subset of SAR202 genotypes^12^. SAR202 bacteria are widely distributed throughout the water column of the ocean, ranging from the surface to the deep ocean trenches^6,15,18^. They become relatively more abundant in the deeper ocean compared to the surface water^6,14^. Previous metagenomic studies rarely exceeded 20 Gb per sample in the sequencing depth of seawater samples^13,19–21^. Considering the diversity of SAR202 in the ocean, this sequencing coverage may not be high enough to recover low abundant SAR202 genomes. Deeper sequencing coverage has the potential to explore new taxa and construct more high-quality metagenomes, providing a comprehensive understanding of the metabolic and ecological functions of microbes in the ocean.

The current genome taxonomy database (GTDB) database contains approximately 400 representative SAR202 metagenome-assembled genomes (MAGs), 92.5% mainly derived from the upper ocean samples (above the oxygen minimum zone, OMZ), such as Tara ocean samples^22^. Although SAR202 bacteria can make up a significant part of the deep ocean microbial community, the number of available MAGs from the deep ocean is still limited compared to the upper ocean (below OMZ).

In this study, we applied deep metagenomic sequencing (180 Gb per sample) to analyze six depth samples at the BATS station. Our sequencing depth surpasses that of most previous studies by at least 10-fold. Our study greatly expands the number of SAR202 genomes or MAGs. It enables us to identify novel SAR202 groups/subgroups in the ocean, which could provide sufficient SAR202 genomes to understand their vertical distribution, metabolic diversity, and unique ecological niches in the ocean.

## Results and Discussion

### Deep metagenomic sequencing recovered more MAGs per sample

**Fig. 1:**
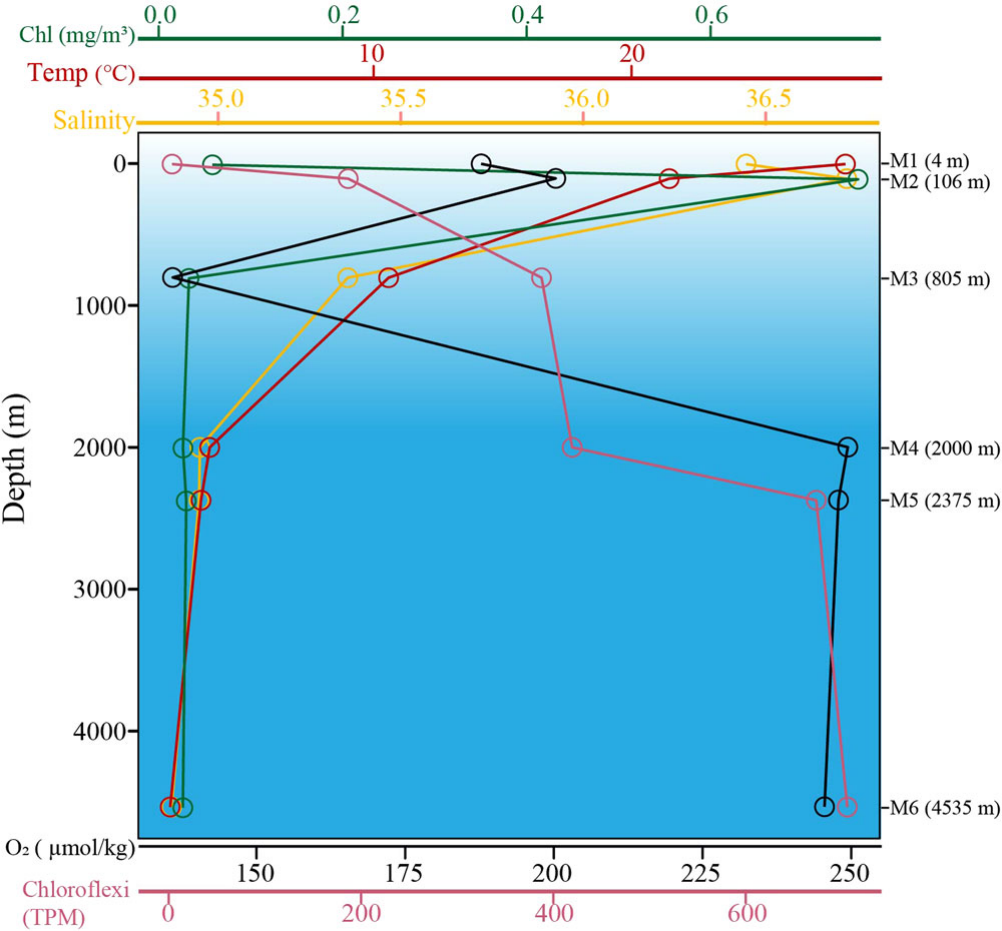
The vertical profile of the key hydrological and biological data at the BATS station. Samples (M1-M6) were collected at six different depths (4, 106, 805, 2000, 2375, and 4535 m, respectively).

The vertical physicochemical profile of the BATS sampling site is shown in Fig. 1. Briefly, the chlorophyll a concentration reached the maximum (0.74 mg/m^3^) at 106 m depth. Dissolved oxygen reached its minimum (136 µmol/kg) at 805 m depth, and increased to 245–249 µmol/kg in the bathypelagic zone. Temperature declined progressively from 28.3 °C to 3.7 °C within the first 2000 m depth and stabilized at approximately 3 °C from 2000 to 4500 m depth. Salinity decreased from 36.7 to 34.8 PSU from the surface to deeper waters (Fig. 1). Deep metagenome sequencing was performed on six samples collected at six different depths (M1-M6) at the BATS station. At least 2 Gb assembly contigs ( > 2,000 bp) were obtained for each sample (Table 1).

**Table 1:** Deep metagenome sequencing information in the BATS station

Sample ID	Depth (m)	Raw data (Gb)	Clean data (Gb)	Assembly Contigs ( > 500)	Assembly Contigs ( > 2000)	N50	ORFs	Total MAGs	Chloroflexota MAGs
M1	4	180	175	19.4 Gb	2.23 Gb	1280	2097129	73	0
M2	106	180	173	15.2 Gb	2.22 Gb	1111	2177040	114	2
M3	805	180	176	20.1 Gb	2.48 Gb	1036	2352332	171	26
M4	2000	180	174	12.9 Gb	2.7 Gb	1361	2664498	209	40
M5	2373	180	177	12.7 Gb	3.27 Gb	1564	3187673	321	72
M6	4535	180	176	10.3 Gb	3.09 Gb	1678	3018008	360	77

Binning of these metagenome data yielded a total of 1248 non-redundant medium/high-quality MAGs (Completeness > 50%, Contamination < 10%, ANI < 95%) (Table 1, Supplementary data 1). On average, 208 MAGs were obtained per sample. The Tara Oceans study yielded 2631 MAGs from 234 samples (averaging 11 MAGs per sample)^23^. The Malaspina expedition recovered 236 MAGs from 58 bathypelagic samples (averaging 4 MAGs per sample)^19^. The sequencing depth of our BATS metagenome (180 Gb per sample) was significantly higher than that of the Tara Ocean ( ~ 30 Gb per sample) and Malaspina database ( ~ 3.4 Gb per sample). The deep metagenomic sequencing applied in this study enabled us to assemble a high number of MAGs. It was a common practice for earlier studies to sequence microbial metagenomes with a sequencing capacity of 10–20 Gb per sample. However, this sequencing depth is not sufficient to recover rare species in the microbial community^24,25^. Our study used the traditional short-read shotgun metagenomics with a capacity of 180 Gb per sample, which is ca. 10-fold higher than most of the earlier studies. Such a sequencing depth allowed us to identify more novel microbial species.

The number of recovered MAGs gradually increased with depth at the BATS station (from 73 MAGs at the surface to 360 MAGs at 4535 m depth) (Table 1). The lower recovery of MAGs in the upper ocean could be related to the vertical distribution of microbes in the ocean. It has been known that microbial diversity and abundance generally decrease from the surface to the deep ocean^26,27^. It will require a higher sequencing depth to assemble more high-quality MAGs in the upper ocean than in the deep ocean. Except for two MAGs identified in the DCM layer, all Chloroflexota MAGs were recovered from the dark ocean, ranging from 805 to 4535 m. Specifically, 26 Chloroflexota MAGs were found at 805 m, 40 at 2000 m depth, 72 at 2373 m depth, and 74 at 4535 m depth (Table 1), reflecting their increasing abundance towards the deeper ocean^14,15,28^.

### A high proportion of unclassified species and genera in the MAGs recovered from BATS

**Fig. 2:**
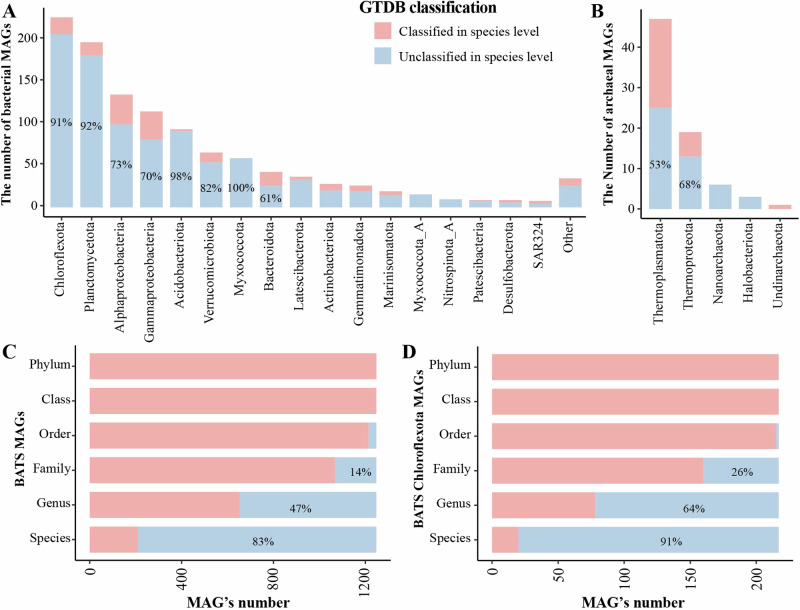
The proportion of classified and unclassified taxa of bacterial, archaeal and Chloroflexota MAGs recovered from BATS based on the GTDB classification. The upper panel shows the number of BATS MAGs in bacteria (**A**) and archaea (**B**). The lower panel shows the GTDB classification in different taxonomic levels (species, genus, family, and order) from 1248 BATS MAGs (**C**) and 217 Chloroflexota MAGs (**D**). The unclassified taxa are presented in blue color, and the classified in red color.

Among the 1248 BATS MAGs, 1172 bacterial and 76 archaeal MAGs were recovered, as shown in Fig. 2A, B. Chloroflexota has the highest number of MAGs (217 MAGs), followed by Planctomycetota (205 MAGs), Alphaproteobacteria (140 MAGs), Gammaproteobacteria (119 MAGs), and Acidobacteriota (97 MAGs). Notably, 83% of these MAGs represent novel species, and 47% are attributed to previously unidentified genera (Fig. 2C). Interestingly, 91% of the 217 Chloroflexota MAGs are novel species and 64% are new genera (Fig. 2D), suggesting a large proportion of Chloroflexota in the ocean remains unexplored.

**Fig. 3:**
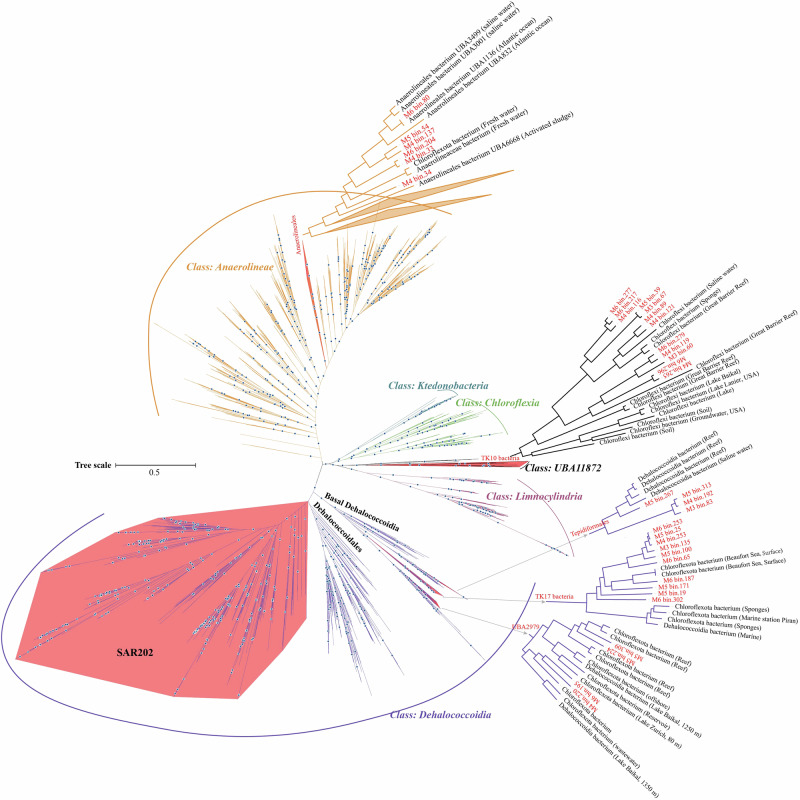
Phylogenomic classification of Chloroflexota based on a total of 1722 Chloroflexota genomes retrieved from the GTDB database (15-Apr-2022). The maximum likelihood tree was inferred from the concatenation of 120 proteins. The 217 Chloroflexota MAGs recovered from the BATS station were labeled in red, and all known representative Chloroflexota genomes were labeled in black. Different classes of Chloroflexota were shown in different colors. The detailed taxonomy is shown in Supplementary data 2.

**Fig. 4:**
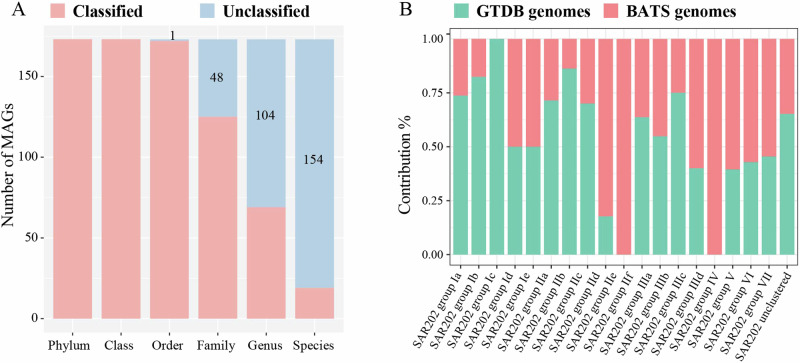
The contribution of 173 BATS SAR202 MAGs. The GTDB classification of 173 BATS SAR202 MAGs (**A**) and their contribution proportion for current SAR202 groups/subgroups number (**B**).

The phylogenomic analysis of the Chloroflexota reveals that SAR202 is a deeply branched monophyletic group that radiates within the Chloroflexota, and SAR202 is a sister group next to Dehalococcoidales (Fig. 3), which is consistent with prior identifications of SAR202 bacteria^15^. Notably, 173 MAGs recovered from BATS were assigned to the SAR202 clade (Fig. 3), in which 154 were classified as new species, 104 as new genera, 48 as new families, and one as new order (Fig. 4A). Our data greatly expanded the phylogenomic tree of SAR202, particularly within the lesser abundant groups IV, V, VI, and VII (Fig. 4B), indicating that deep metagenome sequencing used in this study substantially augments the diversity of SAR202 bacteria in the ocean.

### Phylogenomic diversity of SAR202

**Fig. 5:**
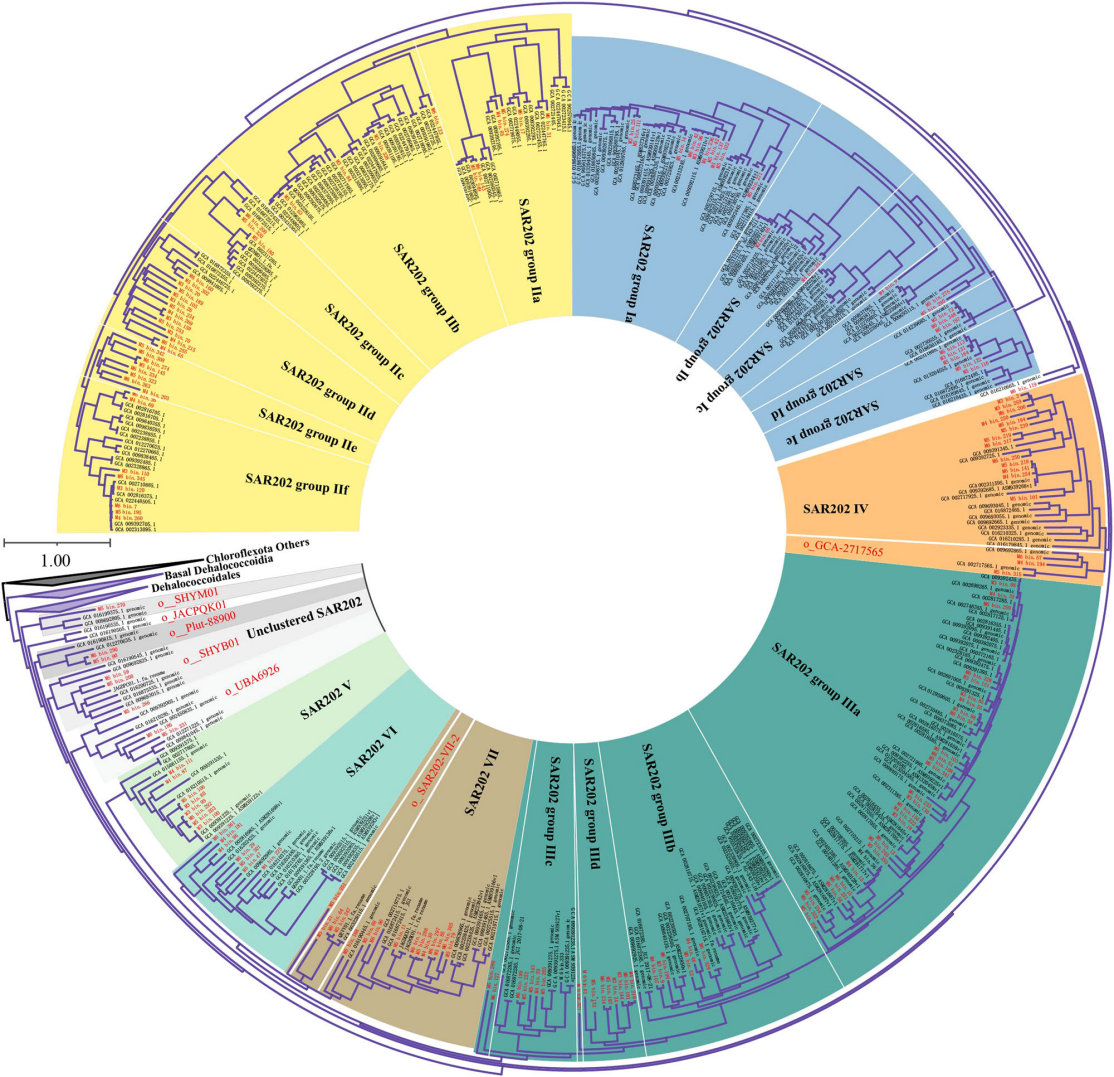
Phylogenomic classification of SAR202 bacteria. This tree is an expansion of the SAR202 branch in Fig. 3. The seven known SAR202 groups were labeled with different colors. The unclustered SAR202 between SAR202 group V and Dehalococcoidales were labeled with different gray shades.

Seven groups (I-VII) of SAR202 bacteria have been reported in earlier studies and correspond to different GTDB orders^12,15^. Interestingly, eight of our SAR202 MAGs do not belong to these seven SAR202 groups. Instead, they fell into five distinct branches between SAR202 group V and Dehalococcoidales (Fig. 5). Each of these five branches is associated with a unique GTDB order name, o_SHYM01, o_JACPQK01, o_Plut-88900, o_SHYB01, and o_UBA6926 (Supplementary data 2), suggesting the presence of unclassified SAR202 members. This Unclustered SAR202 group appears to emerge earlier than the seven known SAR202 groups (Fig. 5). The taxonomic and evolutionary position of these Unclustered SAR202 genomes remains to be confirmed when more genome sequences become available.

Our SAR202 MAGs covered all the known SAR202 groups and subgroups, except for SAR202 group Ic (Fig. 5). We added two new subgroups (Id and Ie) to group I and one new subgroup (IIId) to group III (Fig. 5). Groups I and III each only contained 3 subgroups (Ia-Ic and IIIa-IIIc) previously^15^. We first divided SAR202 group II into six subgroups (IIa-IIf) and subgroups IId and IIe mainly contain our MAGs recovered from BATS. Two new lineages were added to groups VII and IV, respectively, and they include two GTDB orders (o_ SAR202-VII-2 and o_GCA-2717565).

### Vertical distribution of SAR202 bacteria in the global ocean

**Fig. 6:**
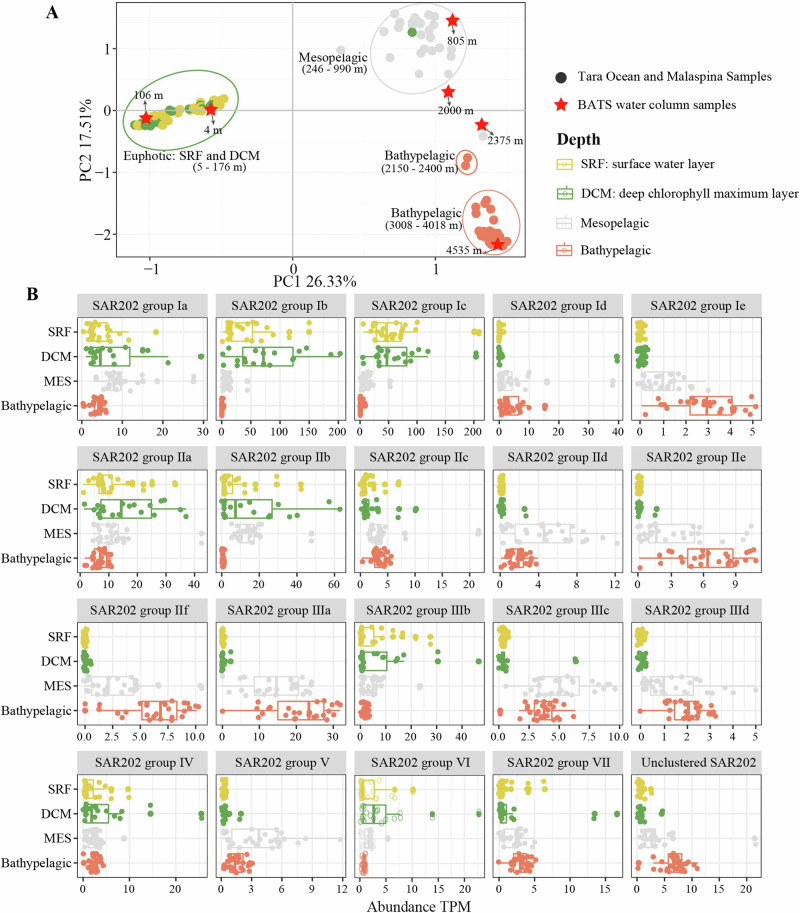
Niche partitioning of SAR202 bacteria in different depths of oceans. The Principal Component Analysis (PCA) shows the clustering of SAR202 communities collected from four different depths (surface, DCM, mesopelagic, and bathypelagic water) (**A**). The relative abundance of SAR202 groups or subgroups at four different depths (surface, DCM, mesopelagic, and bathypelagic) of the world’s oceans (**B**). Dots present the samples from Tara Oceans samples (from surface to 990 m) Malaspina deep samples (from 2150 to 4018 m), and BATS (from 4 to 4535 m). Four different colors of dots represent 4 different depths.

The PCA analysis illustrates that the SAR202 community contains distinct clusters corresponding to the major depths which include the euphotic (SRF and DCM), mesopelagic, and bathypelagic zones (Fig. 6A). Our study indicated that the SAR202 community varies with depth in the world ocean, which is consistent with previous studies in the marine trenches and Caspian Sea water column^14–16^. We plotted the occurrence of 20 newly defined SAR202 subgroups at four major ocean depths (surface, DCM, mesopelagic, and bathypelagic) (Fig. 6B). SAR202 subgroups Id (average 4.1 TPM), Ie (average 1.9 TPM), IIc (average 3.5 TPM), IId (average 3.3 TPM), IIe (average 4.9 TPM), IIf (average 4.5 TPM), IIIa (average 17.5 TPM), IIIc (average 4.5 TPM), and IIId (average 1.7 TPM) are relatively more abundant in the deeper ocean (below 800 m) compared to their abundance in the euphotic ocean which has TPM ranging between 0.001 to 0.9. The abundance of subgroups Ib (average 54.9 TPM), Ic (average 60.9 TPM), IIa (average 12.9 TPM), IIb (average 10.6 TPM), and IIIb (average 5.8 TPM) within group I, II, and III are more prevalent in the photic zone ( > 200 m depth) than that in dark ocean (TPM ranging from 3.2 to 9.2) (Fig. 6B). Group I, II, and III are dominant SAR202 in the ocean^14,15^. An earlier study reported that the SAR202 group I dominates the euphotic ocean^15^. However, we found that some group I subgroups (i.e. Id and Ie) are present in the deep ocean, suggesting that niche partitioning can be different at the subgroup level. Except for subgroup IIIb within group III, most group III subgroups are abundant in the deep ocean, which is consistent with the previous study^15^. The Unclustered SAR202 group is more prevalent in the mesopelagic and bathypelagic ocean than in the euphotic ocean. SAR202 groups IV-VII are present throughout the whole water column and showed less distinguishable vertical patterns compared to groups I-III (Fig. 6). SAR202 groups IV, VI, and VII are abundant in the euphotic water, suggesting that they are more prevalent in the euphotic zone. Such distribution patterns of SAR202 bacteria reflect the ecological diversity and adaptation strategies of microbial life in response to varying environmental factors such as light, temperature, pressure, and nutrient availability within different ocean depths.

### Genomic characteristics of SAR202 groups/subgroups

**Fig. 7:**
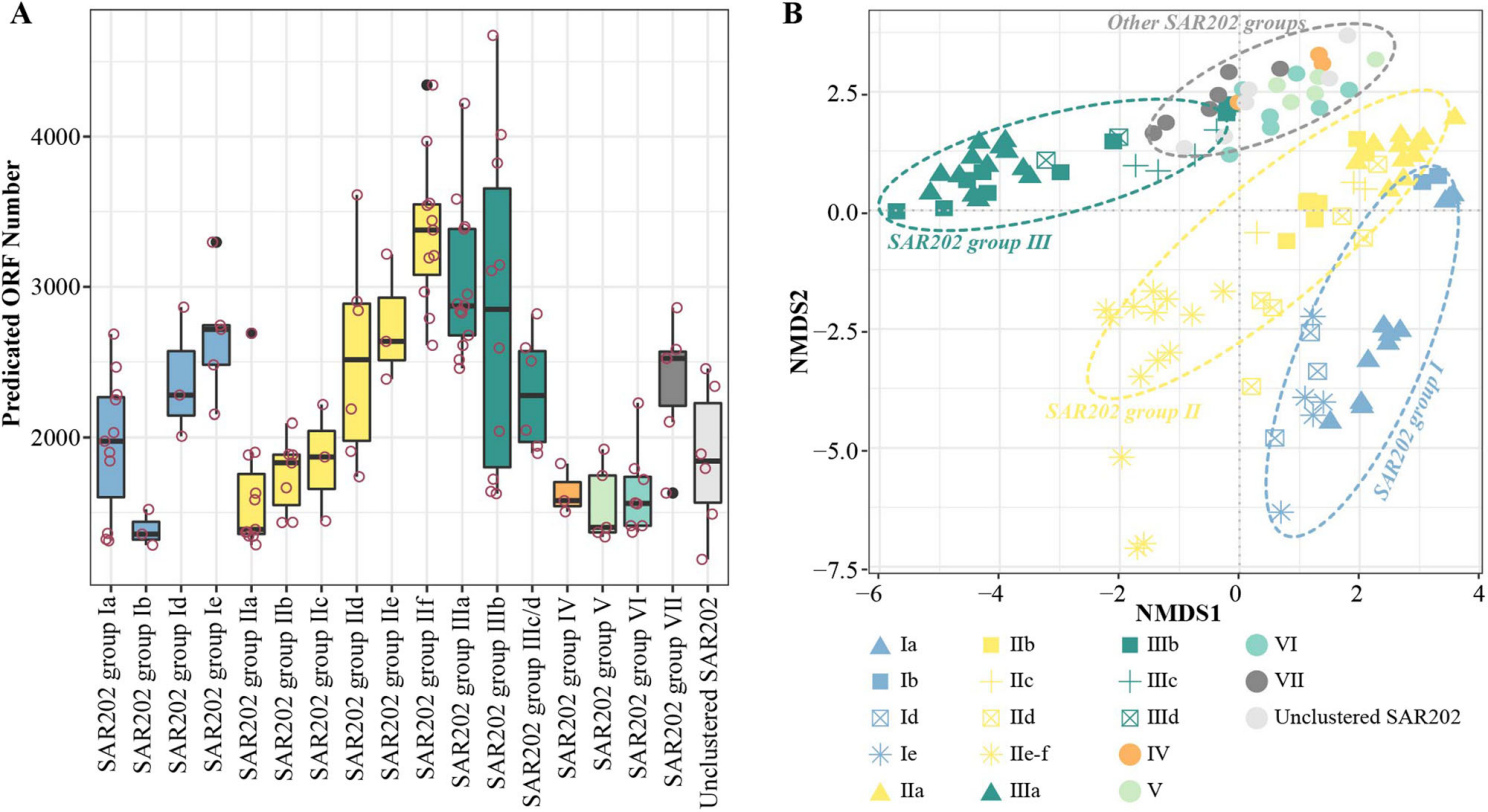
Genetic information in different SAR202 groups/subgroups based on 124 high-quality ( > 90% Completeness) SAR202 genomes derived from GTDB and BATS samples. **A** The number of open reading frames (ORF) across SAR202 groups, and (**B**) Non-metric Multidimensional Scaling (NMDS) analysis of the genomic functional composition of various SAR202 groups/subgroups based on KEGG annotation.

We chose 124 high-quality SAR202 genomes with over 90% completeness from all 471 genomes to analyze their genomic characteristics of groups/subgroups (Supplementary data 3). These genomes consisted of 84 GTDB genomes and 40 BATS genomes, and they covered all known SAR202 groups/subgroups. The deep ocean SAR202 subgroups (Id, Ie, IId, IIe, IIf, IIIa, IIIb, IIIc, IIId) encode ~ 1000 more ORFs than the euphotic subgroups (Ib, Ic, IIa, IIb) (Fig. 7A), suggesting that these SAR202 subgroups in the euphotic ocean may have smaller genome sizes (with fewer genes) compared to those SAR202 subgroups in the deep ocean. Notably, SAR202 subgroup IIIb tends to have a wide range of ORF numbers (Fig. 7A) and is widely distributed in the ocean water column compared to the other subgroups of group III (Fig. 6).

Based on the frequency of KO in each high-quality SAR202, NMDS analysis was used to explore the functional composition similarity of each SAR202 genome (Fig. 7B). Our study shows that the functional composition varies between SAR202 groups/subgroups. A distinct separation in SAR202 groups I, II, III, and other SAR202 groups is evident (Fig. 7B), indicating a functional difference between these SAR202 groups. The function similarity of SAR202 group III is distinct from SAR202 groups I and II based on the gene composition (Fig. 7B), reflecting their distant phylogenomic relationships (Fig. 5). Different groups of SAR202 may contain specific genes needed based on their adaptative natures. For example, a previous study found that the FNNOs genes only appear in SAR202 III, while the enolase genes are widely present in group I^15^.

### Metabolic difference of SAR202 in different depths

**Fig. 8:**
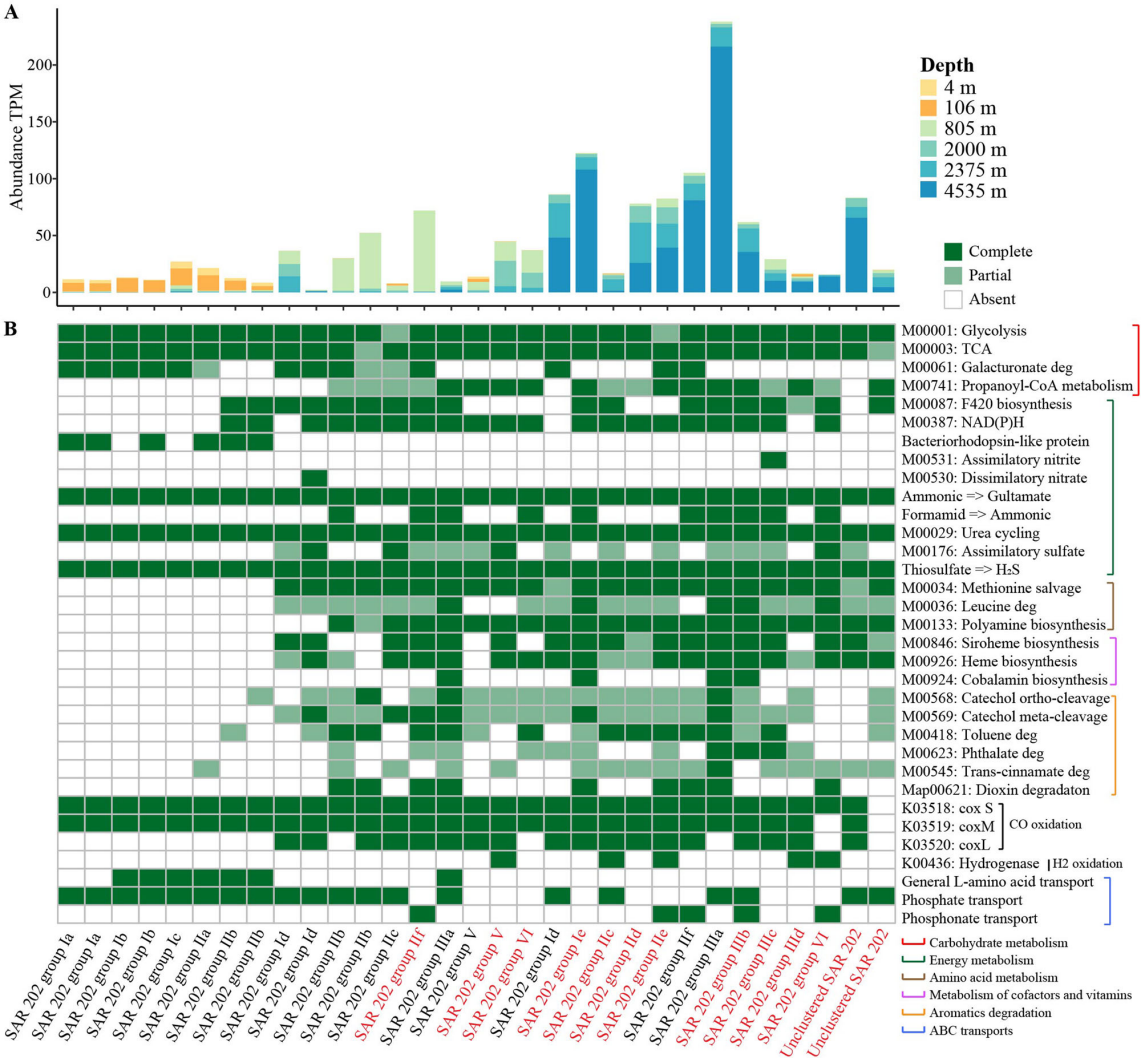
Metabolic characteristics of 31 dominant high-quality SAR202 bacteria in the vertical ocean. The relative abundance in the BATS water column and genome size (**A**), and selected metabolic functions (**B**) of the 31 selected high-quality SAR202 MAGs ( > 90% completeness) which represent different SAR202 groups (except for groups IV and VII). The detailed genome information is shown in Supplementary data 5. Samples from Tara Oceans, Malaspina, and the BATS station were utilized to determine the average abundance of each MAG. The MAGs from BATS were labeled in red.

To elucidate the functional disparities of SAR202 across varying ocean depths, we analyzed 31 genomes out of 124 high-quality SAR202 genomes (Supplementary data 5). These 31 genomes represent relatively more abundant SAR202 groups in the ocean because their average relative abundance is higher than 10 TPM. These genomes were identified in different ocean depths, including eight genomes from the euphotic, ten from the mesopelagic, and thirteen from the bathypelagic zones. Notably, the relative abundance of these genomes varies significantly with depth in the BATS water column (Fig. 8A), underscoring their suitability to represent the change of SAR202 bacteria in different ocean depths.

SAR202 bacteria in the dark ocean exhibit more complex metabolic functions compared to those in the euphotic zone, particularly in the degradation of aromatic compounds (Fig. 8B). Genes associated with the degradation of substances such as catechol, toluene, trans-cinnamate, phthalate, and polyaromatic hydrocarbons including dioxin are prevalent among deep ocean SAR202 bacteria, yet are absent in their euphotic counterparts (Fig. 8B). Our study indicated that deep ocean SAR202 bacteria have notably potential for degrading complex dissolved organic carbon (Fig. 8B). This is consistent with the previous finding that SAR202 bacteria derived from the marine trench and dark ocean are prone to degrade refractory dissolved organic carbon (RDOC)^13,16,17^. More than 95% of DOC in the deep ocean water is RDOC, and it was previously suggested that it can remain in the deep ocean for hundreds to thousands of years^29,30^, although the age of deep-ocean DOM is currently debated^31^ and RDOC turnover times are unknown. The fact that deep ocean SAR202 bacteria have the capability to break down RDOC implies that the RDOC pool in the dark ocean is subjected to bacterial degradation further fueling the debate on RDOC turnover times. Although we do not know the actual degradation rate of RDOC by SAR202, it is plausible that SAR202 bacteria could play an active role in the turnover of the ocean’s RDOC considering their genomic versatility for degrading RDOC.

Deep ocean SAR202 bacteria exhibit enhanced capabilities for synthesizing a wider array of amino acids and cofactors/vitamins compared to their euphotic counterparts (Fig. 8B). Notably, pathways such as leucine degradation, methionine salvage, polyamine biosynthesis, siroheme biosynthesis, heme biosynthesis, and cobalamin biosynthesis are prevalent in the deep ocean SAR202, yet are absent in the euphotic SAR202 (Fig. 8B). In contrast, the general L-amino acid transport system is commonly observed in the euphotic SAR202 but is rare in the deep ocean SAR202, suggesting that the utilization of amino acids directly from seawater could be important to SAR202 in the surface ocean. Interestingly, the cobalamin biosynthesis is enriched in SAR202 group III (Fig. 8B), a phenomenon also seen in SAR202 in the Mariana Trench^16,18^. It appears that all SAR202 bacteria have the potential to assimilate ammonium and urea and be involved in the reduction of thiosulfate to hydrogen sulfide (Fig. 8B). In addition, SAR202 bacteria in the deep ocean have the potential to assimilate sulfate, utilize organic sulfur such as alkanesulfonate, and oxidize sulfite (Fig. 8B, Supplementary data 6). Together, these genomic features suggest that SAR202 bacteria can be important in the ocean’s sulfur cycling. SAR202 bacteria in the bathypelagic ocean layers have the potential to utilize multiple organosulfur compounds and oxidize sulfite^14^. Sulfite oxidation can generate ATP and thus provide an essential energy source for SAR202 in the Ionian Sea 3500 m and Mariana Trench^14,18^. Some deep ocean SAR202 bacteria show the potential for using phosphonate (Fig. 8B), suggesting a metabolic adaptation for utilizing organic phosphorus in the deep ocean.

SAR202 bacteria in the euphotic ocean (subgroups Ia, Ib, IIa, and IIb within groups I and II) encode the bacteriorhodopsin-like genes (Fig. 8B). Bacterioplankton in sunlit oceanic regions commonly possess the proteorhodopsin gene, facilitating additional energy production through a light-driven proton pump^32^. Previous research has confirmed the presence of the proteorhodopsin gene in SAR202 strains retrieved from waters shallower than 150 meters^15^, suggesting the critical role of photic energy utilization in these SAR202 bacteria. Moreover, the predicted galactonate dehydratase (dgoD) gene, a member of the COG4948 paralogs, is prevalent in SAR202 group I (at least 12 dgoD genes per genome), which is far more abundant than other SAR202 groups (Supplementary data 6). This gene cluster is abundant in cultured SAR202 strains from group Ia, known for their capacity to metabolize various carbohydrates^12^. In the euphotic ocean, phytoplankton release polysaccharides which can be rapidly assimilated by bacteria^30,31^. We hypothesize that there is a close ecological interaction between SAR202 bacterioplankton and phytoplankton in the photic zone.

It is noteworthy that the three CO dehydrogenase genes (coxS, coxM, and coxL) are widely present in the deep water SAR202 bacteria, while SAR202 bacteria in the photic zone only contain the coxS and coxM genes but not the coxL gene (Fig. 8B). It has been reported that CO oxidation provides energy which supports microbial growth and survival in the ocean^33^. The cox genes have been found in Chloroflexota^34^ and SAR202^13^. The coxL gene is the large catalytic subunit of dehydrogenase genes. It would be interesting to see if the surface SAR202 bacteria lose the CO oxidation function since they do not encode the coxL gene.

## Conclusion

Deep metagenomic sequencing at the BATS water column has revealed substantial insights into the genomic diversity and metabolic capabilities of SAR202 bacteria across different ocean depths. By recovering a significant number of MAGs, especially from the deeper ocean water, we expanded the phylogenetic diversity of marine SAR202 from 11 to 23 groups/subgroups and nearly doubled the number of SAR202 MAGs in the current metagenome database. We found that SAR202 bacteria (subgroups Id, Ie, IIc, IId, IIe, IIf, IIIa, IIIc, and IIId within groups I, II, and III) in the bathypelagic zone possess enhanced metabolic functions for degrading complex organic compounds and biosynthesizing essential amino acids and cofactors/vitamins. Conversely, SAR202 bacteria (subgroup Ia, Ib, IIa, and IIb) in the euphotic zone harness light-driven processes and interact closely with phytoplankton. The SAR202 bacteria in the surface ocean likely utilize labile organic substrates produced by photosynthetic organisms. On the other hand, deep ocean SAR202 bacteria are more capable of degrading recalcitrant DOC, supporting the previous hypothesis that SAR202 bacteria have the potential to degrade more complex and resistant dissolved organic matter in the deep ocean^13,15^. This research not only highlights the ecological significance of SAR202 bacteria but also sets a foundation for future studies aimed at understanding their specific functions and interactions within marine ecosystems.

## Methods

### Sample and environmental data collection

Six samples were collected from different depths (4, 106, 805, 2000, 2373, and 4535 m depth) at the BATS station (31°40’ N, 64°10’ W) aboard the R/V Atlantic Explorer on August 5–11, 2019. These water samples were labeled M1 to M6, representing surface (M1), deep chlorophyll maximum (DCM) (M2), oxygen minimum zone (OMZ) (M3), and bathypelagic zone (M4 – M6). For each sample, 120 L of seawater was collected using Niskin bottles and prefiltered through a 3 μm pore-size polycarbonate membrane (142 mm in diameter, Pall) with a peristaltic pump. Subsequently, the filtrate was filtered through one 0.22 μm pore-size polycarbonate membrane (142 mm in diameter, Pall). The filters were stored in a −80 °C freezer during the cruise, shipped with liquid N_2_, and stored in a −80 °C freezer in the laboratory until DNA extraction. Microbial cells retained on the 0.22 μm filters (0.22–3 μm) were used for DNA extraction. The CTD profiles obtained environmental data such as temperature, salinity, oxygen, and fluorescence.

### DNA extraction and sequencing

Microbial DNA was extracted from half of the 0.22 μm filter described above following a phenol-chloroform protocol^35^. 200 ng of DNA for each sample was used to prepare the sequencing library. Shotgun sequencing (paired-end 2 × 150 bp) was performed using the Illumina HiSeq2000 platform at the Genome Resource Center, University of Maryland School of Medicine, and ca. 180 Gb of raw data was obtained for each sample. The flowchart of the bioinformatics analysis is shown in Fig. S1. Trimmomatic 0.36^36^ was used to remove low-quality reads (LEADING:10, TRAILING:10, SLID-INGWINDOW:4:20, MINLEN:70). The detailed information is shown in Table 1.

### Metagenome assembly, binning, classification

Each sample was separately assembled using megahit v1.2.9^37^ with the default parameters. QUAST v5.0.2^38^ assessed assembly quality (average N50 = 1,340). Assembled contigs > 2000 bp from each sample were automatically binned into MAGs based on a combination of nucleotide coding frequencies and sequence assembly coverage using Metabat2 v.2.12.1^39^, Maxbin2 v.2.2.4^40^ and CONCOCT v.1.0.0^41^. Contig abundance for binning was obtained by mapping trimmed reads to assemblies using bowtie2^42^ and samtools^43^. Genome bins from each binning tool were aggregated by the metaWRAP: Bin_refinement module^44^. The completeness and contamination of the MAGs were evaluated by CheckM v.1.0.7^45^. All MAGs were finally dereplicated using dRep^46^ with the ANI (Average Nucleotide Identity) cut-off value of ≥ 95% in the secondary ANI comparison. These MAGs were classified with the Genome Taxonomy Database (GTDB) by the GTDB-Tk tool^47^.

### Analysis of Chloroflexota MAGs

Based on GTDB results, a total of 217 Chloroflexota MAGs were obtained from the BATS water column. The average abundance of Chloroflexota MAGs in each sample is calculated by taking the length-weighted average of the MAGs’ contig abundances by salmon v 0.13.1^48^. Open reading frames (ORFs) of Chloroflexota MAGs were predicted by Prokka v1.14.6^49^. Predicted genes from each MAGs were annotated against the Kyoto Encyclopedia for Genes and Genomes (KEGG) and eggnog database^50,51^ using the diamond v0.9.14^52^ (E-value = 1 × 10^−^^6^).

### Phylogenetic tree analysis

The 217 BATS MAGs and all 1502 representative Chloroflexota genomes of the GTDB database (as of 15-Apr-2022)^22^ were used to construct a phylogenomic tree, using 120 core genes which were identified using GTDB-Tk v0.1.3^47^. These core genes were aligned and concatenated using the gtdbtk aline modules with default parameters (If a genome had a low number of markers identified, it will be excluded from the analysis at this step). IQ-Tree was used to infer single-gene phylogenies with the following parameters (-bb 1000)^53^. Resulting maximum likelihood phylogenetic trees were utilized to analyze the classification of 217 BATS Chloroflexota MAGs. The phylogenomic tree was visualized using iTOL^54^. The categories of MAGs were primarily determined by the placement of the genomes in the phylogenetic trees. We identified these novel SAR202 subgroups based on the observation that single branches contain more than three SAR202 genomes and ANI is lower than 70% when compared to nearby genomes.

### Predicting heliorhodopsin genes

A total of 585 reference sequences of heliorhodopsin (HeR) were downloaded from NCBI. After manually examining these sequences, BLAST v2.12.0^55^ was applied to build a HeR gene protein database (makeblastdb) to annotate predicated HeR protein. The predicted genes in each Chloroflexota MAGs were converted into protein sequences and mapped to the HeR gene protein database with blastp (E-value = 1 × 10^−^^6^) to obtain the HeR gene information.

### Evaluating the abundance of SAR202 bacteria in the oceans

A total of 471 SAR202 genomes (including our 173 SAR202 MAGs and 298 downloading SAR202 genomes from GTDB) were used to investigate the vertical community structure of the SAR202 community in the world’s ocean (0–4535 m depth). The raw sequences samples (0.2–3 μm) from the Tara Ocean^56^ and all Malaspina samples^19^ were downloaded from the European bioinformatics institute (EBI). These samples are mainly distributed in the Atlantic, Indian, and Pacific oceans—the detailed information is in Supplementary data 3. Salmon v 0.13.1^48^ was applied to calculate the coverage of all SAR202 contigs in each sample. Coverage tables were acquired to assess the TPM (transcripts per million) abundance of each contig in each sample. The TPM abundance of each bin in each sample was calculated by taking the length-weighted average of the bins’ contig abundances with script split_salmon_out_into_bins.py in the metawrap^44^.

### Selection of high-quality SAR202 MAGs and comparative genomics

CheckM v.1.0.7^45^ was applied to evaluate the quality of all SAR202 genomes. We picked high-quality SAR202 genomes based on their high completeness ( > 90%) and low contamination ( < 10%). These genomes were used to compare genomic features between different groups/subgroups. We then further selected 31 SAR202 genomes that are abundant ( > 10 TPM) based on their occurrence frequency in the database of Tara Ocean, Malaspina, and BATS samples. These high abundance SAR202 genomes are selected to represent SAR202 genomes in different depths, such as euphotic, OMZ, and bathypelagic ocean. These genomes are annotated in the KEGG and eggnog database^50,51^. Metabolic comparison was performed based on the presence and absence of specific KEGG modules.

### Statistics and analysis

All the calculations and plots were performed in an R environment (version 4.3.3). All bar and dot charts were plotted using the ggplot2 (version 3.5.0) package^57^. According to the TPM abundance of SAR202 in the Tara Ocean, Malaspina, and BATS samples, the vegan package (version 2.6-4) was used for the PCA (principal component analysis) analysis^58^. This shows the distribution of SAR202 bacteria in the world’s oceans. High-quality genomes of SAR202 bacteria produced 3922 different KO (KEGG Orthology) via KEGG annotation. A table was created by calculating the frequency of KOs in each SAR202 genome, assigning a value of 0 if the genome lacked the corresponding KO. The NMDS (non-metric multidimensional scaling) analysis (using the bray-curtis dissimilarity index) was applied to analyze the genomic composition of SAR202 bacteria using the gene frequency data. The distance in NMDS represents the functional similarity of MAGs.

### Reporting summary

Further information on research design is available in the Nature Portfolio Reporting Summary linked to this article.

### Supplementary information

Supplementary Material

Description of Additional Supplementary Materials

Supplementary Data 1-6

Reporting summary

## Data Availability

The metagenome-sequencing reads can be accessed at the NCBI database (project no. PRJNA911943). The sequence and contigs information are shown in Table 1. All binned genomes obtained from the BATS station are available in the figshare database^59^ (10.6084/m9.figshare.25020137).

## Code availability

For metagenome data analyses, shell scripts (for quality control, assembling, binning, etc.) are used at Zenodo^60^ (10.5281/zenodo.12609004), as detailed in the “Methods” section of the paper.

## References

[1] Giovannoni, S. J., Rappé, M. S., Vergin, K. L. & Adair, N. L. 16S rRNA genes reveal stratified open ocean bacterioplankton populations related to the Green Non-Sulfur bacteria. *Proc. Natl. Acad. Sci. USA* **93**, 7979–7984 (1996). 10.1073/pnas.93.15.7979 PMC38860 8755588

[2] Fuhrman, J. A. & Davis, A. A. Widespread Archaea and novel Bacteria from the deep sea as shown by 16S rRNA gene sequences. *Mar. Ecol. Prog. Ser.* **150**, 275–285 (1997).

[3] Morris R, Rappé M, Urbach E, Connon S, Giovannoni S (2004). Prevalence of the Chloroflexi-related SAR202 bacterioplankton cluster throughout the mesopelagic zone and deep ocean. Appl. Environ. Microbiol..

[4] DeLong EF (2006). Community genomics among stratified microbial assemblages in the ocean’s interior. Science.

[5] Schattenhofer M (2009). Latitudinal distribution of prokaryotic picoplankton populations in the Atlantic Ocean. Environ. Microbiol..

[6] Varela MM, Van Aken HM, Herndl GJ (2008). Abundance and activity of Chloroflexi‐type SAR202 bacterioplankton in the meso‐and bathypelagic waters of the (sub) tropical Atlantic. Environ. Microbiol..

[7] Chandler DP, Brockman FJ, Bailey T, Fredrickson JK (1998). Phylogenetic diversity of archaea and bacteria in a deep subsurface paleosol. Microb. Ecol..

[8] Gich F, Garcia-Gil J, Overmann J (2001). Previously unknown and phylogenetically diverse members of the green nonsulfur bacteria are indigenous to freshwater lakes. Arch. Microbiol..

[9] Hentschel U (2002). Molecular evidence for a uniform microbial community in sponges from different oceans. Appl. Environ. Microbiol..

[10] Mehrshad M (2018). Hidden in plain sight—highly abundant and diverse planktonic freshwater Chloroflexi. Microbiome.

[11] Urbach E (2001). Unusual bacterioplankton community structure in ultra‐oligotrophic Crater Lake. Limnol. Oceanogr..

[12] Lim Y, Seo J-H, Giovannoni SJ, Kang I, Cho J-C (2023). Cultivation of marine bacteria of the SAR202 clade. Nat. Commun..

[13] Landry, Z., Swan, B. K., Herndl, G. J., Stepanauskas, R. & Giovannoni, S. J. SAR202 genomes from the dark ocean predict pathways for the oxidation of recalcitrant dissolved organic matter. *MBio.* **8**, 00413–00417 10.1128/mbio (2017). 10.1128/mBio.00413-17 PMC5395668 28420738

[14] Mehrshad M, Rodriguez-Valera F, Amoozegar MA, López-García P, Ghai R (2018). The enigmatic SAR202 cluster up close: shedding light on a globally distributed dark ocean lineage involved in sulfur cycling. ISME J..

[15] Saw, J. H. et al. Pangenomics analysis reveals diversification of enzyme families and niche specialization in globally abundant SAR202 bacteria. *MBio.* **11**, 02975–02919 10.1128/mbio (2020). 10.1128/mBio.02975-19 PMC6946804 31911493

[16] Wei Z, Li Q, Lu R, Zheng P, Wang Y (2022). In situ genomics and transcriptomics of SAR202 subclusters revealed subtle distinct activities in deep-sea water. Microorganisms.

[17] Liu R (2022). Novel Chloroflexi genomes from the deepest ocean reveal metabolic strategies for the adaptation to deep-sea habitats. Microbiome.

[18] Wei Z-F, Li W-L, Huang J-M, Wang Y (2020). Metagenomic studies of SAR202 bacteria at the full-ocean depth in the Mariana Trench. Deep Sea Res. Part I: Oceanogr. Res. Pap..

[19] Acinas SG (2021). Deep ocean metagenomes provide insight into the metabolic architecture of bathypelagic microbial communities. Commun. Biol..

[20] Lim Y, Yang S-J, Kang I, Cho J-C (2023). Metagenomic data from surface seawater of the east coast of South Korea. Sci. data.

[21] Lima LF (2023). Coral and seawater metagenomes reveal key microbial functions to coral health and ecosystem functioning shaped at reef scale. Microb. Ecol..

[22] Parks DH (2022). GTDB: an ongoing census of bacterial and archaeal diversity through a phylogenetically consistent, rank normalized and complete genome-based taxonomy. Nucleic acids Res..

[23] Tully BJ, Graham ED, Heidelberg JF (2018). The reconstruction of 2631 draft metagenome-assembled genomes from the global oceans. Sci. data.

[24] Bickhart DM (2022). Generating lineage-resolved, complete metagenome-assembled genomes from complex microbial communities. Nat. Biotechnol..

[25] Jin H (2022). Hybrid, ultra-deep metagenomic sequencing enables genomic and functional characterization of low-abundance species in the human gut microbiome. Gut Microbes.

[26] Alves Junior N (2015). Microbial community diversity and physical–chemical features of the Southwestern Atlantic Ocean. Arch. Microbiol..

[27] Namirimu T (2023). Microbial Diversity of Deep-sea Sediments from Three Newly Discovered Hydrothermal Vent Fields in the Central Indian Ridge. Ocean Sci. J..

[28] Morris RM (2005). Temporal and spatial response of bacterioplankton lineages to annual convective overturn at the Bermuda Atlantic Time‐Series Study Site. Limnol. Oceanogr..

[29] Jiao N (2010). Microbial production of recalcitrant dissolved organic matter: long-term carbon storage in the global ocean. Nat. Rev. Microbiol..

[30] Osterholz H, Niggemann J, Giebel H-A, Simon M, Dittmar T (2015). Inefficient microbial production of refractory dissolved organic matter in the ocean. Nat. Commun..

[31] Gonsior M, Powers L, Lahm M, McCallister SL (2022). New perspectives on the marine carbon cycle–the marine dissolved organic matter reactivity continuum. Environ. Sci. Technol..

[32] DeLong EF, Beja O (2010). The light-driven proton pump proteorhodopsin enhances bacterial survival during tough times. PLoS Biol..

[33] Cordero PR (2019). Atmospheric carbon monoxide oxidation is a widespread mechanism supporting microbial survival. ISME J..

[34] Martin-Cuadrado A-B, Ghai R, Gonzaga A, Rodriguez-Valera F (2009). CO dehydrogenase genes found in metagenomic fosmid clones from the deep Mediterranean Sea. Appl. Environ. Microbiol..

[35] Kan J, Wang K, Chen F (2006). Temporal variation and detection limit of an estuarine bacterioplankton community analyzed by denaturing gradient gel electrophoresis (DGGE). Aquat. Microb. Ecol..

[36] Bolger AM, Lohse M, Usadel B (2014). Trimmomatic: a flexible trimmer for Illumina sequence data. Bioinformatics.

[37] Li D (2016). MEGAHIT v1. 0: a fast and scalable metagenome assembler driven by advanced methodologies and community practices. Methods.

[38] Gurevich A, Saveliev V, Vyahhi N, Tesler G (2013). QUAST: quality assessment tool for genome assemblies. Bioinformatics.

[39] Kang DD, Froula J, Egan R, Wang Z (2015). MetaBAT, an efficient tool for accurately reconstructing single genomes from complex microbial communities. PeerJ.

[40] Wu Y-W, Simmons BA, Singer SW (2016). MaxBin 2.0: an automated binning algorithm to recover genomes from multiple metagenomic datasets. Bioinformatics.

[41] Alneberg J (2014). Binning metagenomic contigs by coverage and composition. Nat. methods.

[42] Langmead B, Salzberg SL (2012). Fast gapped-read alignment with Bowtie 2. Nat. methods.

[43] Li H (2009). The sequence alignment/map format and SAMtools. Bioinformatics.

[44] Uritskiy GV, DiRuggiero J, Taylor J (2018). MetaWRAP—a flexible pipeline for genome-resolved metagenomic data analysis. Microbiome.

[45] Parks DH, Imelfort M, Skennerton CT, Hugenholtz P, Tyson GW (2015). CheckM: assessing the quality of microbial genomes recovered from isolates, single cells, and metagenomes. Genome Res..

[46] Olm MR, Brown CT, Brooks B, Banfield JF (2017). dRep: a tool for fast and accurate genomic comparisons that enables improved genome recovery from metagenomes through de-replication. ISME J..

[47] Chaumeil P-A, Mussig AJ, Hugenholtz P, Parks DH (2022). GTDB-Tk v2: memory friendly classification with the genome taxonomy database. Bioinformatics.

[48] Patro R, Duggal G, Love MI, Irizarry RA, Kingsford C (2017). Salmon provides fast and bias-aware quantification of transcript expression. Nat. methods.

[49] Seemann T (2014). Prokka: rapid prokaryotic genome annotation. Bioinformatics.

[50] Huerta-Cepas J (2019). eggNOG 5.0: a hierarchical, functionally and phylogenetically annotated orthology resource based on 5090 organisms and 2502 viruses. Nucleic acids Res..

[51] Kanehisa M, Goto S, Kawashima S, Okuno Y, Hattori M (2004). The KEGG resource for deciphering the genome. Nucleic acids Res..

[52] Buchfink B, Xie C, Huson DH (2015). Fast and sensitive protein alignment using DIAMOND. Nat. methods.

[53] Nguyen L-T, Schmidt HA, Von Haeseler A, Minh BQ (2015). IQ-TREE: a fast and effective stochastic algorithm for estimating maximum-likelihood phylogenies. Mol. Biol. evolution.

[54] Letunic I, Bork P (2021). Interactive Tree Of Life (iTOL) v5: an online tool for phylogenetic tree display and annotation. Nucleic acids Res..

[55] Ye J, McGinnis S, Madden TL (2006). BLAST: improvements for better sequence analysis. Nucleic acids Res..

[56] Sunagawa S (2015). Structure and function of the global ocean microbiome. Science.

[57] Wickham, H., Chang, W. & Wickham, M. H. Package ‘ggplot2’. Create elegant data visualisations using the grammar of graphics. Version 2, 1-189 (2016).

[58] Oksanen J (2007). The vegan package. Community Ecol. package.

[59] He, C. Metagenome-assembled genomes in the BATS water column [Data set]. Figshare 10.6084/m9.figshare.25020137 (2024).

[60] He, C. Deep metagenomic sequencing unveils novel SAR202 lineages and their vertical adaptation in the ocean. Zenodo 10.5281/zenodo.12609004 (2024).10.1038/s42003-024-06535-538997445

