# Supplementary material for: Deep metagenomic sequencing unveils novel SAR202 lineages and their vertical adaptation in the ocean
Source: Commun Biol. 2024 Jul 12;7:853. doi: 10.1038/s42003-024-06535-5 (PMC11245477; doi:10.1038/s42003-024-06535-5)
Supplement: Supplementary file 1 — Supplementary Material [file 42003_2024_6535_MOESM1_ESM.pdf]

Deep metagenomic sequencing unveils novel SAR202 lineages and their vertical adaptation in the ocean

Changfei He[1,2,3], Daniel Fucich[3], Ana Sosa[3], Hualong Wang[3,4], Jinjun Kan[5], Jihua Liu[2], Yongle Xu[2], Nianzhi Jiao[1], Michael Gonsior[6], Feng Chen[3, *]

[1] State Key Laboratory of Marine Environmental Science, College of Ocean and Earth Sciences, Carbon Neutral Innovation Research Center and Fujian Key Laboratory of Marine Carbon Sequestration, Xiamen University, Xiamen 361102, PR China

[2] Institute of Marine Science and Technology, Shandong University, Qingdao 266237, China

[3] Institute of Marine and Environmental Technology, University of Maryland Center for Environmental Science, Baltimore, Maryland 21202, USA

[4] College of Marine Life Sciences, Frontiers Science Center for Deep Ocean Multispheres and Earth System, and Key Lab of Polar Oceanography and Global Ocean Change, Ocean University of China, Qingdao, China

[5] Microbiology Division, Stroud Water Research Center, Avondale, PA 19311, USA

[6] Chesapeake Biological Laboratory, University of Maryland Center for Environmental Science, Solomons, Maryland 20783, USA

*Correspondence: chenf@umces.edu

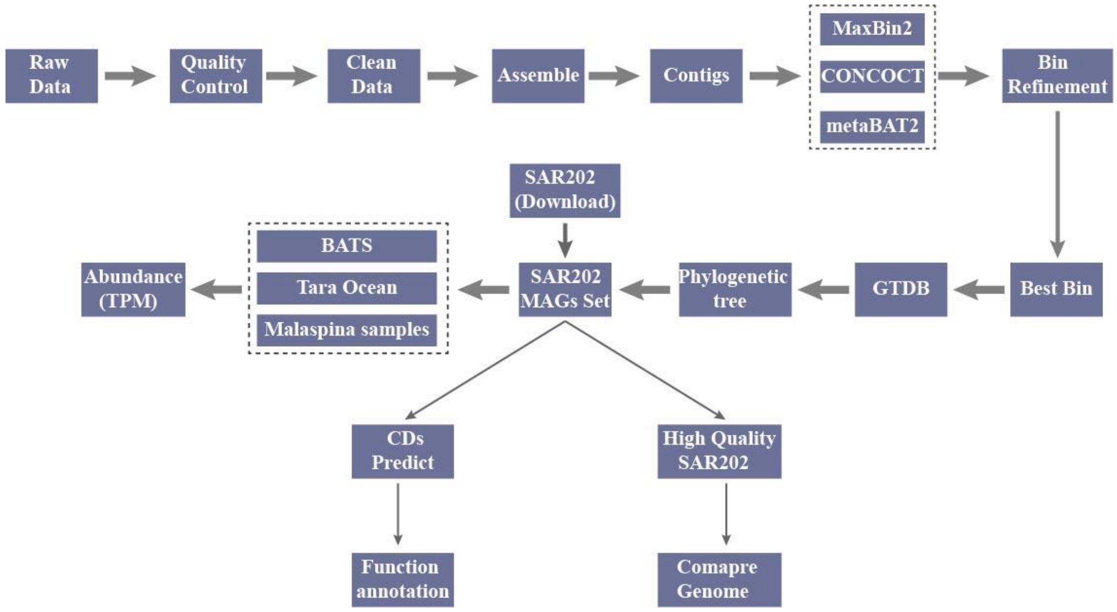

Fig. S1 Illustration of the flowchart of the bioinformatics analysis
