# Supplementary material for: Deep metagenomic sequencing unveils novel SAR202 lineages and their vertical adaptation in the ocean
Source: Commun Biol. 2024 Jul 12;7:853. doi: 10.1038/s42003-024-06535-5 (PMC11245477; doi:10.1038/s42003-024-06535-5)
Supplement: Supplementary file 2 — Description of Additional Supplementary Materials [file 42003_2024_6535_MOESM2_ESM.pdf]

Description of Additional Supplementary Files

File name: Supplementary Data 1

Description: 1248 MAGs recovered from BATS station

File name: Supplementary Data 2

Description: The information of 217 Chloroflexi MAGs.

File name: Supplementary Data 3

Description: The detailed information of downloaded Tara ocean and Malaspina samples.

File name: Supplementary Data 4

Description: Information for 124 highquality SAR202 genomes.

File name: Supplementary Data 5

Description: The information of selected 31 SAR202 genomes.

File name: Supplementary Data 6

Description: The top 50 genes in the 31 selected SAR202 genomes
